# Localization and Sub-Cellular Shuttling of HTLV-1 Tax with the miRNA Machinery

**DOI:** 10.1371/journal.pone.0040662

**Published:** 2012-07-10

**Authors:** Rachel Van Duyne, Irene Guendel, Zachary Klase, Aarthi Narayanan, William Coley, Elizabeth Jaworski, Jessica Roman, Anastas Popratiloff, Renaud Mahieux, Kylene Kehn-Hall, Fatah Kashanchi

**Affiliations:** 1 Department of Molecular and Microbiology, National Center for Biodefense & Infectious Diseases, George Mason University, Manassas, Virginia, United States of America; 2 Department of Microbiology, Immunology, & Tropical Medicine, The George Washington University Medical Center, Washington, D.C., United States of America; 3 Molecular Virology Section, Laboratory of Molecular Microbiology, National Institute of Allergy and Infectious Diseases, National Institutes of Health, Bethesda, Maryland, United States of America; 4 Department of Anatomy and Regenerative Biology, The George Washington University, Washington, D.C., United States of America; 5 Retroviral Oncogenesis Team, INSERM-U758 Virologie Humaine, Lyon, France; University of Hong Kong, Hong Kong

## Abstract

The innate ability of the human cell to silence endogenous retroviruses through RNA sequences encoding microRNAs, suggests that the cellular RNAi machinery is a major means by which the host mounts a defense response against present day retroviruses. Indeed, cellular miRNAs target and hybridize to specific sequences of both HTLV-1 and HIV-1 viral transcripts. However, much like the variety of host immune responses to retroviral infection, the virus itself contains mechanisms that assist in the evasion of viral inhibition through control of the cellular RNAi pathway. Retroviruses can hijack both the enzymatic and catalytic components of the RNAi pathway, in some cases to produce novel viral miRNAs that can either assist in active viral infection or promote a latent state. Here, we show that HTLV-1 Tax contributes to the dysregulation of the RNAi pathway by altering the expression of key components of this pathway. A survey of uninfected and HTLV-1 infected cells revealed that Drosha protein is present at lower levels in all HTLV-1 infected cell lines and in infected primary cells, while other components such as DGCR8 were not dramatically altered. We show colocalization of Tax and Drosha in the nucleus *in vitro* as well as coimmunoprecipitation in the presence of proteasome inhibitors, indicating that Tax interacts with Drosha and may target it to specific areas of the cell, namely, the proteasome. In the presence of Tax we observed a prevention of primary miRNA cleavage by Drosha. Finally, the changes in cellular miRNA expression in HTLV-1 infected cells can be mimicked by the add back of Drosha or the addition of antagomiRs against the cellular miRNAs which are downregulated by the virus.

## Introduction

Human T-lymphotropic virus type 1 (HTLV-1) was originally discovered in 1980, identified as the first human retrovirus, and currently infects more than 20 million people worldwide [1]–[5]. HTLV-1 is the etiologic agent of adult T-cell leukemia/lymphoma (ATLL) and HTLV-1-associated myelopathy/tropical spastic paraparesis (HAM/TSP) in infected individuals. Oncogenesis is due primarily to the viral transactivator protein, Tax, a 40-kDa phosphoprotein that regulates not only viral transcription, but acts to manipulate host cellular functions such as cell cycle progression, apoptosis, chromatin remodeling, and other signal transduction pathways [6]–[8]. Recently, much interest has developed in elucidating the cross-talk between tumor development and HTLV-1 infection as it relates to the innate host response, in particular the small RNA regulatory network.

Human microRNA (miRNA) sequences derived from the genome have the ability to silence cellular genes and are currently considered a primary host immune defense against cellular invaders such as pathogens and viruses. In a host cell, miRNAs are the product of the RNA interference (RNAi) pathway, a regulatory and innate defense mechanism that is conserved in eukaryotes. This pathway utilizes short non-coding RNA sequences of 18–21 nucleotides to bind to mRNA sequences with complementary homology, subsequently restricting the translation of these transcripts [9]. Following RNA Pol II transcription of a gene, the Pri-miRNA consists of a series of RNA hairpins protruding from an RNA message with a 5′cap and poly-A tail. This Pri-miRNA is cleaved by a microprocessor complex of nuclear proteins, Drosha, an RNase II endonuclease, and DCGR8 (Pasha), an RNA-binding protein, to form a stem and loop RNA-structure called Pre-miRNA. This Pre-miRNA is shuttled out of the nucleus into the cytoplasm via Exportin5 and is further processed by an additional RNase III enzyme, Dicer, which cleaves the hairpin into a short miRNA duplex. These miRNA then associate with the RNA induced silencing complex (RISC), composed of Ago2 and TRBP proteins, which then aids in miRNA-mediated target recognition. This guide effector protein complex assists in either degrading the targeted message or preventing its translation [10]. The dysregulation of this pathway is highly evident across a variety of cancers and viruses, including HIV-1, HTLV-1, Influenza, HCV, Ebola, Vaccinia, PFV-1, LACV, Adenovirus, and SARS-CoV although the mechanisms of action for most of these viruses remains to be determined [11]–[27]. Indeed, cellular miRNAs are able to silence endogenous retroviruses, sequences which typically share a high degree of homology to present day retroviruses, such as HTLV-1 and HIV-1.

HTLV-1 Tax acts to transactivate the viral long terminal repeat (LTR) through Tax-responsive elements (TREs) in the U3 region. This occurs through transcriptional induction of TREs, posttranslational modifications of TRE-binding factors, and binding with transcription factors. Tax is known to interact with the transcription factors CREB, serum-responsive factor (SRF), and NF-κB as well as with the cell cycle related proteins Cyclin D2 and D3, mitotic checkpoint regulators (MAD1), cyclin-dependent kinases (cdks), cdk inhibitors p16^INK4a^ and p21/waf1, and p53 [5], [28]–[43]. Phosphorylation of Tax is necessary for Tax localization in nuclear bodies as well as activation of cellular gene expression through the NF-κB pathway. Tax activates HTLV-1 transcription through CREB and three CRE enhancer sequences on the LTR. Tax also interacts with CBP/p300, which is involved in the formation of the preinitiation complex as well as the p300/CBP-associated factor, P/CAF, which also plays a role in active HTLV-1 gene transcription [37], [44]–[46]. Taken together, HTLV-1 Tax is heavily involved in direct interactions with critical cellular proteins that control not only viral gene expression but also cellular genes involved in tumor formation.

We have previously shown that Tax interacts directly with the cellular Rb (Retinoblastoma) protein and targets Rb for degradation via the proteasome pathway, resulting in a decrease in Rb protein expression in HTLV-1 infected cells and a dysregulation of the cell cycle [47]. Due to the nature of Tax to manipulate and control cellular proteins such as Rb and overall cell cycle progression, we became interested in determining the effect of HTLV-1 infection and Tax expression on other cellular proteins regulating oncogenesis, including the RNAi pathway. HTLV-1 encodes an additional regulatory protein, called Rex, which has recently been implicated in having RNA silencing suppressor activity. Specifically, Rex interacts with both RNA and Dicer, suppressing Dicer’s enzymatic activity [15]. Recently, Tax has been shown to specifically mediate the downregulation of cellular miRNAs which are associated with the regulation of chromatin remodeling factors [48]. Specifically, Tax downregulated miR-149 and miR-873, both of which directly target p300 and P/CAF [48]. This suggests that HTLV-1 infection and its viral transactivators may play a role in dysregulation of the RNAi pathway. Here we show that HTLV-1 infection in the presence of Tax, significantly downregulates Drosha protein expression. We show the *in vitro* interaction between Tax and Drosha, both in the nucleus and in complex in the presence of proteasome inhibitors. We observe a downregulation of Drosha functionality in the presence of Tax as measured by primary miRNA cleavage. Finally, the changes in cellular miRNA expression in HTLV-1 infected cells can be mimicked in uninfected cells supplementing excess functional Drosha or the addition of antagomiRs against the cellular miRNAs which are downregulated by the virus.

## Results

### HTLV-1 Downregulates Core Components of the RNAi Machinery

**Figure 1:**
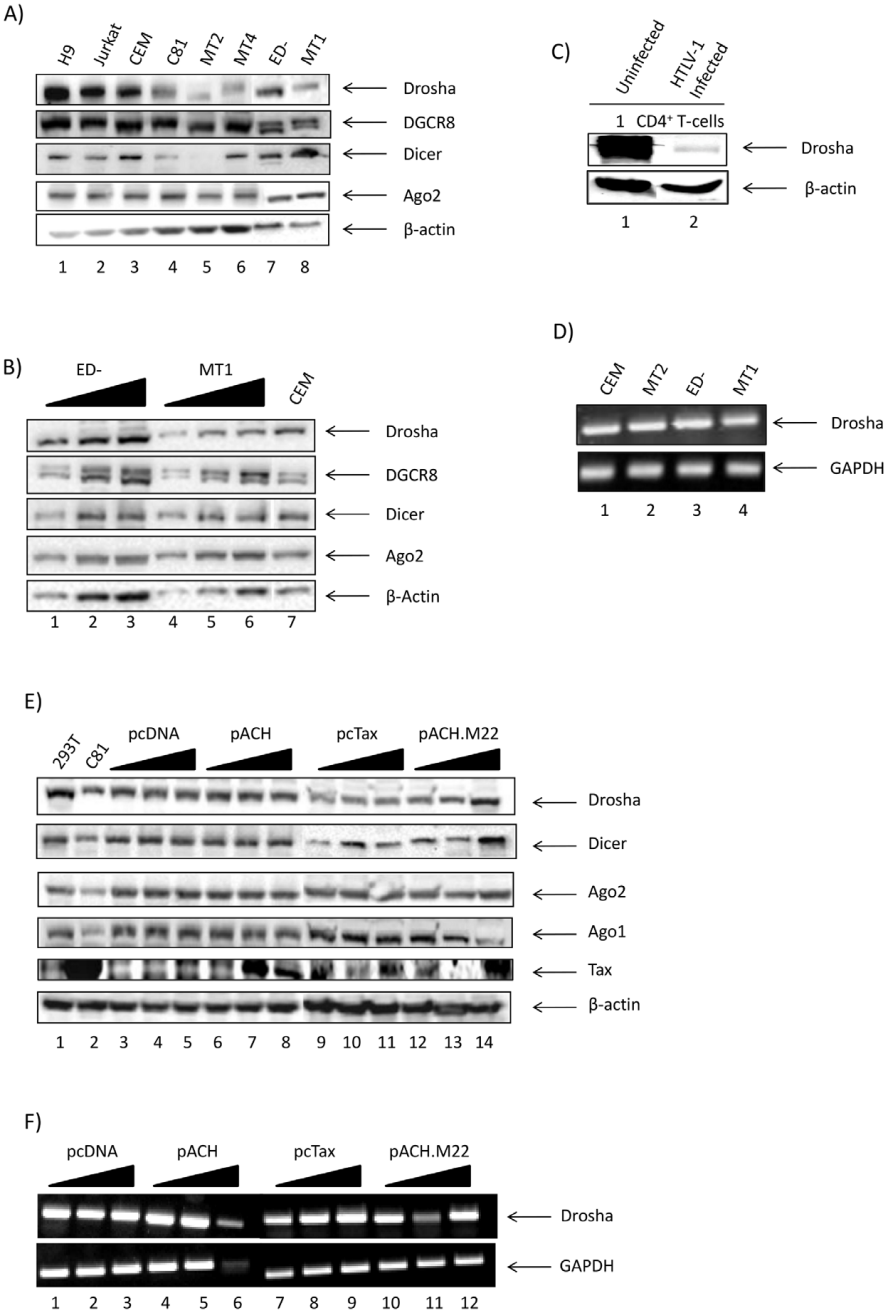
The RNAi machinery is dysregulated with HTLV-1 infection. **A**) Uninfected (H9, Jurkat, CEM), HTLV-1 infected, Tax-positive (C81, MT2, MT4), and HTLV-1 infected, Tax-negative (ED-, MT1) T-cell lines were screened for endogenous protein levels of Drosha, DGCR8, Dicer, and Ago2. Seventy-five micrograms of total lysates were used for western blots. Multiple bands for DGCR8 are indicative of isoforms at 86 and 65 kDa. β-Actin serves as a loading control. **B**) Total lysates of HTLV-1 infected, Tax-negative cell lines ED- and MT1 were titrated (50, 75 and 100 µg) and screened for endogenous protein levels of Drosha, DGCR8, Dicer, and Ago2. Multiple bands for DGCR8 are indicative of isoforms at 86 and 65 kDa. β-Actin serves as a loading control. **C**) Whole cell lysates (50 µg) from HTLV-1 infected primary CD4^+^ T-cells were run on a 10% Tris-glycine SDS-PAGE gel and western blotted for Drosha. Data is representative of three independent experiments. β-Actin serves as a loading control. **D**) Total RNA was isolated with TRIzol from CEM, MT2, ED-, and MT1 cell lines. RT-PCR was performed for cellular Drosha transcript levels. GAPDH serves as a housekeeping gene loading control. **E**) Uninfected 293T cells were transfected with pcDNA (0.1, 1.0, and 10 µg), pACH (0.1, 1.0, and 10 µg), pcTax (0.1, 1.0, and 10 µg), or pACH.M22 (0.1, 1.0, and 10 µg) and were screened for protein levels of Drosha, Dicer, Ago2, Ago1, and Tax. Seventy-five micrograms of total lysates was used for western blots. β-Actin serves as a loading control. **F**) Total RNA was isolated with TRIzol from the transfected cells in panel E. RT-PCR was performed for cellular Drosha transcript levels. GAPDH serves as a housekeeping gene loading control.

Tax acts as the HTLV-1 viral transactivator protein by interacting with various cellular and viral proteins including transcription factors, kinases, and chromatin remodeling proteins, ultimately altering cellular pathways such as apoptosis and the cell cycle to control viral replication and oncogenesis [6]–[8]. Recently, retroviruses have been shown to control the cellular RNAi pathway in order to evade viral inhibition and also to hijack both the enzymatic and catalytic components of the miRNA machinery. This viral response assists in promoting an active viral infection, or a latent state of infection [11], [48]–[49]. Indeed, other retroviruses such as HIV-1 have been shown to impart a specific suppression of Dicer expression in monocyte-derived-macrophages as compared to T-cells [50]. In order to examine the role HTLV-1 infection has on possible control of the RNAi pathway, we initially screened for the protein levels of core components of the RNAi machinery in HTLV-1 infected cells as compared to uninfected cells. Total protein levels of Drosha, DGCR8, Dicer, Ago2, and β-Actin were detected in three uninfected T-cell lines, H9, Jurkat, and CEM, as well as in three HTLV-1 infected, Tax-positive T-cell lines, C81, MT2, and MT4 (Figure 1A, lanes 1–3 and lanes 4–6). Drosha protein levels were significantly downregulated in all of the HTLV-1 infected, Tax-positive cells tested when compared to the uninfected cells. Specifically, Drosha is decreased by 62.6% in C81 cells, 97.3% in MT2 cells, and 91.5% in MT4 cells when compared to CEM cells (normalized to β-Actin). Dicer protein was also slightly downregulated in C81 cells, but more so in MT2 cells when compared to CEM cells (normalized to β-Actin). Conversely, DGCR8 and Ago2 showed no significant changes in protein level amongst all cell lines screened, suggesting that the downregulation seen for Drosha and Dicer is specific and not a consequence of the entire pathway being inhibited. Interestingly, the protein levels of the core components of the RNAi machinery were also detected in two HTLV-1 infected, Tax-negative cell lines (Figure 1A, lanes 7, 8). Drosha was downregulated by 77.1% in MT1 cells and 51.3% in ED- cells as compared to CEM cells (normalized to β-Actin). We titrated both MT1 and ED- cells in order to determine if this downregulation was an artifact and indeed, with increasing concentrations of protein extract (50, 75, and 100 µg), Drosha levels also increase (Figure 1B), suggesting that the relative decrease in Drosha is less in HTLV-1 infected, Tax-negative cells as compared to Tax-positive cells. We reproduced these results in HTLV-1 infected CD4^+^ primary T-cells (Figure 1C), where Drosha levels are dramatically downregulated as compared to uninfected cells (lane 2 compared to lane 1). In order to rule out the possibility that the loss of Drosha protein is due to a decrease in transcript levels, we performed an RT-PCR of total mRNA isolated from CEM, MT2, ED-, and MT1 cells for Drosha transcripts. In Figure 1D, we show that the Drosha transcript levels are unchanged as compared to GAPDH across all four of these cell lines.

Next, we attempted to mimic this downregulation of Drosha and Dicer by introducing both Tax and HTLV-1 into uninfected 293T cells and screening for core RNAi machinery components. 293T cells were transfected with three concentrations (0.1, 1.0, 10 µg) of plasmids expressing pACH (full-length HTLV-1 clone), pcTax, pACH.M22 (mutated *tax* gene), or an empty vector control. The pACH.M22 plasmid contains a double amino acid substitution at positions 130 and 131 and eliminates the ability of Tax to transactivate the NF-κB pathway [51]–[54]. Cells were collected 48 hours post-transfection and were western blotted for the presence of Drosha, Dicer, Ago2, Ago1, Tax and β-Actin. Figure 1E shows endogenous protein levels of core RNAi machinery components in 293T cells transfected with pcDNA and other vectors. Comparing Drosha proteins levels in cell expressing pACH, pcTax, and pACH.M22 to the pcDNA control (lanes 6, 7, 8 and 9, 10, 11 and 12, 13, 14 to lanes 2, 3, 4, respectively) results in a 27.5% decrease in pACH (10 µg) expressing cells (lane 8), a 67.3% decrease in pcTax (10 µg) expressing cells (lane 11) and only a 10.4% decrease in pACH.M22 (10 µg) expressing cells. These results imply that in the pACH.M22 mutant Tax expressing cells, we observe close to endogenous levels of Drosha, indicating the downregulation of Drosha may be the result of a functional Tax. Overall, relative expression levels of Dicer, Ago2, and Ago1 reproduce the data from Figure 1A. The Tax western blot serves as a positive control for Tax expression (lanes 2 and 6–14). The β-Actin western serves as a positive loading control. Again we confirmed that this decrease in Drosha protein levels is due to Tax expression and not due to an effect on total transcript levels (Figure 1F). Collectively, these data indicate that HTLV-1 infection in the presence of Tax downregulates the RNAi enzyme Drosha in chronically infected cell lines, transfected cells, and primary cells infected with HTLV-1 virus.

### Colocalization of Tax with Drosha in the Nucleus

**Figure 2:**
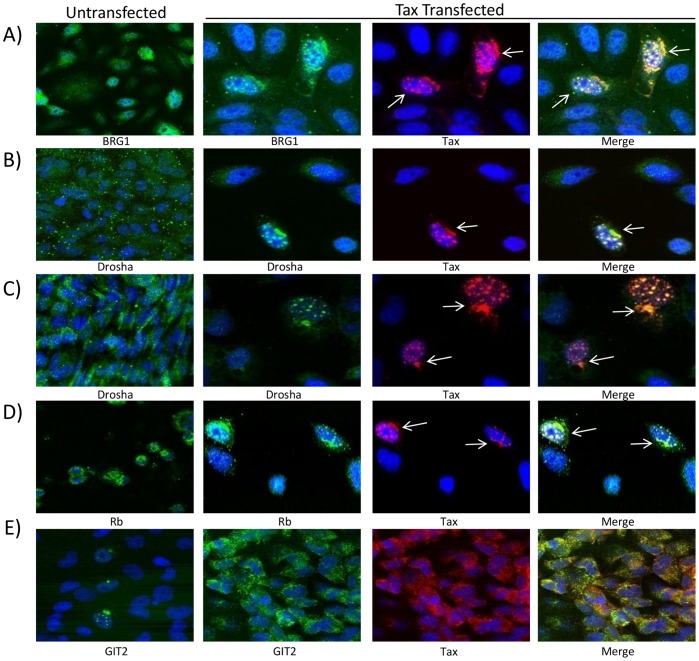
Nuclear colocalization of HTLV-1 Tax with cellular proteins. HeLa cells were grown on coverslips in the presence or absence of HTLV-1 Tax. Forty-eight hours post transfection of pcTax (5 µg), cells were stained for BRG1 (**A**), Drosha (**B, C**), Rb (**D**), and GIT2 (**E**) and were visualized with confocal microscopy. Cellular protein staining is represented in green, Tax staining in red, and yellow foci represent colocalization of dual stained cells. Nuclei were stained with DAPI (blue). GIT2 serves as a cytoplasmic protein control.

The viral protein Tax has been well documented as interacting directly with multiple critical cellular proteins that control oncogenesis. We sought to determine whether or not Tax interacts with Drosha in order to downregulate and/or signal the Drosha protein for possible degradation. To investigate the potential interaction of Tax and Drosha, we transfected pcTax into HeLa cells and stained the cells 48 hours post-transfection for confocal imaging. We labeled both untransfected and Tax-transfected HeLa cells with antibodies against BRG1, Drosha, Rb, GIT2, and Tax. BRG1 and Rb serve as positive Tax-interacting proteins as we have previously shown their intracellular interaction [1], [47]. BRG1 is constitutively expressed in HeLa cells in the absence of Tax, however post-Tax transfection, BRG1 is found almost exclusively in the nucleus and colocalizes with Tax (Figure 2A). Similarly, Rb is heterogeneously expressed in HeLa cells, however colocalizes with Tax in the nucleus of Tax-expressing cells (Figure 2D). Interestingly, in Tax expressing cells, Drosha is almost exclusively found in the nucleus at specific foci colocalizing with Tax (Figures 2B, C). In order to address possible nonspecific shuttling between the nucleus and the cytoplasm due to transfection conditions or overexpression of Tax, we utilized GIT2 to serve as a negative control for a protein that does not shuttle to the nucleus or colocalize in the presence of Tax (Figure 2E). Therefore, our imaging data show that Tax and Drosha colocalize in the nucleus of Tax-expressing cells.

### Drosha is Found in Complex with Tax

**Figure 3:**
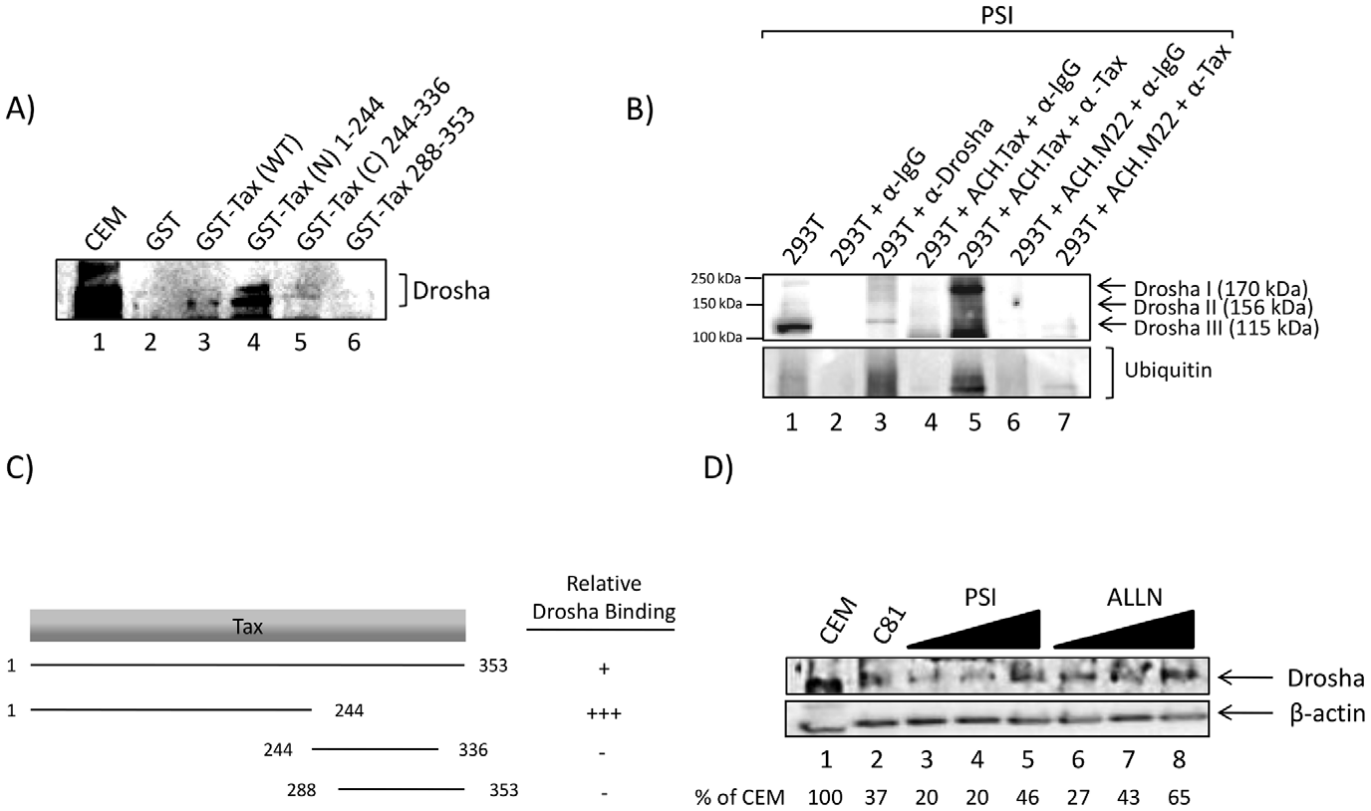
HTLV-1 Tax interacts with Drosha *in vitro*. **A**) HeLa whole cell lysates (1 mg) were incubated with purified GST-fusion proteins (∼5 µg) bound to Glutathione-Sepharose beads: GST-Alone, GST-Tax wild type, GST-Tax N-terminus, 1–244, GST-Tax C-terminus, 244–336, and GST-Tax truncated, 288–353. Beads were washed gently, proteins eluted, and western blotted for the presence of Drosha. CEM whole cell extract serves as a positive Drosha control. **B**) 293T cell lysates were prepared from cells alone or cells transfected with pACH.Tax (5 µg) or pACH.M22 (5 µg) in the presence of the proteasome inhibitor PSI (10 µM). Approximately 1 mg of lysate was incubated with α-IgG, α-Drosha, or α-Tax overnight at 4°C. The next day, 50 µl of a 30% Protein A + G slurry was used to IP Drosha or Tax and subsequently western blotted for the presence of Drosha and Ubiquitin. Multiple isoforms of Drosha as detected by the antibody are indicated as Drosha I (170 kDa), Drosha II (156 kDa), and Drosha III (115 kDa). **C**) Graphical depiction of the GST-Tax constructs and the regions which they span for the Tax protein. Relative Drosha binding is based on densitometry counts. **D**) C81 cells were treated with the proteasome inhibitors PSI and ALLN at increasing concentrations (0.1, 1.0, and 10 µM) for 48 hours. Cell lysates were western blotted for the presence of Drosha. β-Actin serves as a loading control. Drosha recovery as a percentage of CEM Drosha levels is indicated from densitometry counts normalized to β-Actin using ImageJ.

The full length Tax protein contains a variety of domains that impart specific protein-protein interactions as well as interactions with sub-cellular components. The N-terminus of Tax contains both a nuclear localization signal (NLS) as well as a zinc-finger domain which is involved with interactions with cellular transcription factors [31], [49], [55]–[57]. Specifically, this domain of Tax is responsible for Tax-mediated CREB transactivation. The C-terminus of Tax is responsible for nuclear-cytoplasmic shuttling and contains a Golgi localization motif, a secretion motif, an LXCXE-like motif, and a PDZ-binding domain [5], [47], [58]–[64]. Additionally, the region of Tax between the N- and C-termini includes two leucine zipper motifs used for dimerization as well as activation of NF-κB [5], [58]–[61]. We have previously shown that Tax is capable of binding to cellular Rb via its LXCXE-like motif [47]. To identify the domain of Tax that binds to Drosha, we performed an *in vitro* binding assay using full-length Tax and various fragments covering the entire Tax protein fused to GST. Figure 3A shows the results of a GST-Tax pulldown from HeLa whole cell extracts, followed by a western blot for the presence of Drosha. Drosha bound to wild type Tax, but much stronger binding was observed at the N-terminal domain that contained sequence 1–244. This is partly because the C-terminus may fold back and contain inhibitory activity in an *in vitro* binding assay. We have also confirmed the interaction of Tax and Drosha *in vitro* with an immunoprecipitation (IP). We transfected 293T cells with the HTLV-1 clone pACH.Tax (5 µg) or pACH.M22 (5 µg) mutant Tax in the presence of the proteasome inhibitor PSI (10 µM). Our rational for using a proteasome inhibitor was to be able to isolate complexes that were available for biochemical analysis prior to the targeted degradation via the proteosome pathway. We collected cells 48 hours post transfection and lysed whole cell extracts (∼1 mg) for an overnight IP with α-Tax, α-Drosha, or α-IgG antibodies. The following day, Protein A + G beads were incubated with the extracts for 2 hours, the beads were washed with low salt buffer, proteins eluted in Laemmli buffer, and were subsequently western blotted for the presence of Drosha. As shown in Figure 3B, Drosha can be successfully IPed out of cells expressing wild type Tax with α-Tax in the presence of a proteasome inhibitor (lane 5). 293T cells alone serve as a positive control for the Drosha western blot (lane 1) as well as a positive control for the Drosha IP/western blot (lane 3). Interestingly, the mutant ACH.M22 Tax is unable to interact with Drosha (lane 7). This Tax mutant is only faintly ubiquitinylated and can’t interact with the proteasome, therefore indicating that the interaction of Tax and Drosha and the subsequent degradation requires interaction with the proteasome [65]. The α-Drosha antibody used for the western blot recognizes all three isoforms of Drosha, corresponding to the molecular weights of 170, 156, and 115 kDa, which accounts for the multiple Drosha bands. However, the strongest Tax binding is to the 170 kDa form. A ubiquitin western blot is included as an indication that Drosha is being ubiquitinated in the presence of Tax, however, not in the presence of a Tax mutant (compare lane 5 to lane 7). A summary of relative Drosha binding affinity to Tax constructs from multiple experiments is depicted in Figure 3C. Based on these data, Tax binds to Drosha via the N-terminus (aa 1–244), given that the strongest binding was shown to occur within this region, and can also be identified in complex with Drosha. Collectively, these data indicate that Tax and Drosha interact and can be found in complex *in vitro*.

Previously, we have shown that the binding of Tax and Rb results in a targeted degradation of Rb via the proteasome pathway [47]. To investigate whether Tax utilizes a similar mechanism to decrease Drosha protein levels, we designed experiments using proteasome inhibitors. We treated HTLV-1 infected C81 cells with proteasome inhibitors PSI and ALLN (0.1, 1.0, 10 µM). At 48 hours post-treatment, the cells were collected and western blotted for the presence of Drosha. Figure 3D shows the western blot of Drosha normalized to β-Actin. Both PSI and ALLN treated cells showed an increase in Drosha protein levels concurrent with increasing concentration of proteasome inhibitor. Indeed, the 10 µM-treated cells show almost a 30% increase in Drosha protein levels compared to C81 cells alone. All treated cells exhibited no toxicity across three independent experiments (data not shown). This data further indicates that Drosha protein levels can be rescued from suppression by HTLV-1 infection with proteasome inhibitors, suggesting that Drosha is targeted by Tax to be degraded via the proteasomal pathway. Collectively, these data point to the regulation of Drosha by direct binding to the N-terminus of Tax and further degradation by the proteasomal pathway.

### Tax Prevents Primary miRNA Cleavage by Drosha

**Figure 4:**
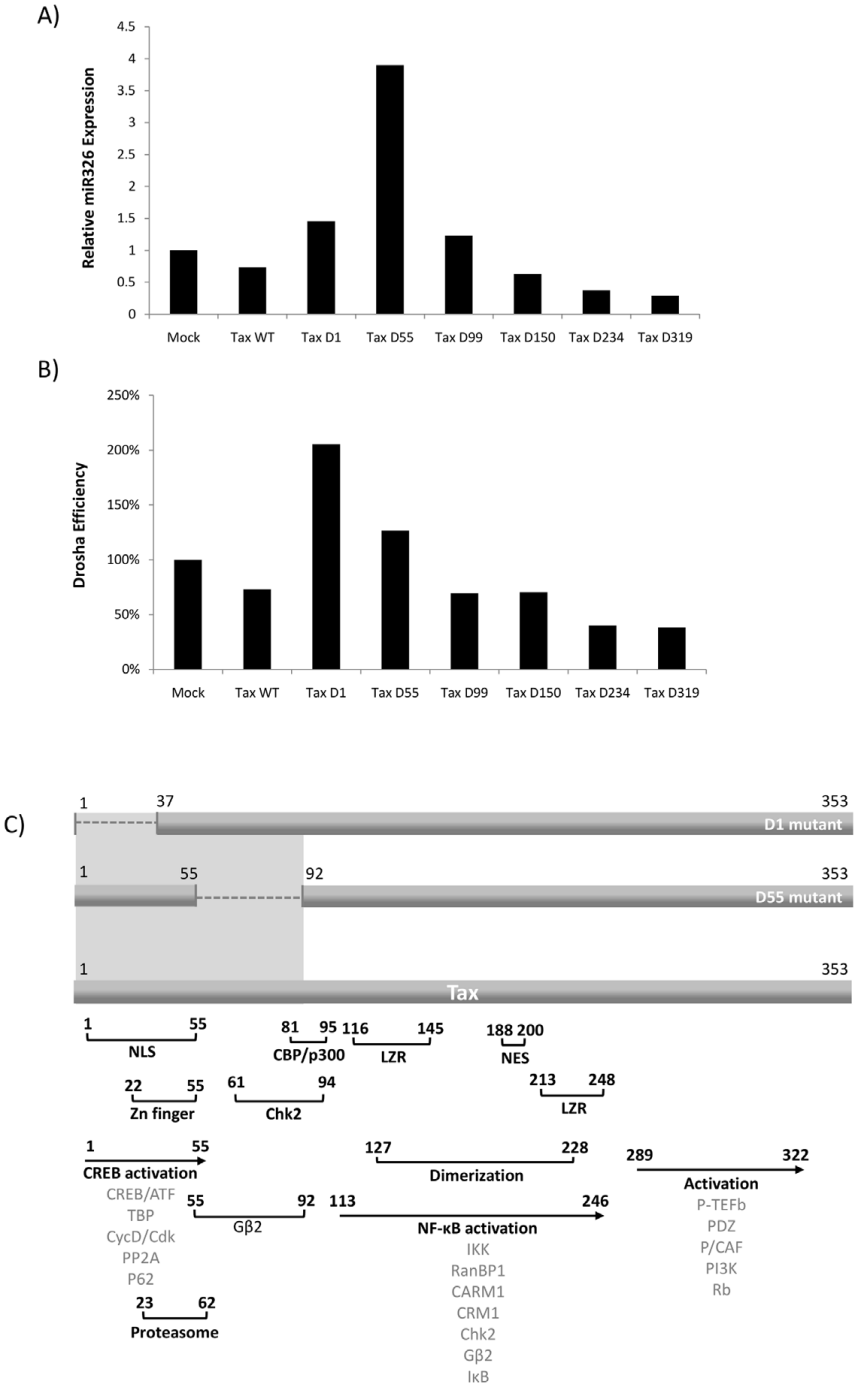
Tax prevents primary miRNA cleavage by Drosha. 293T cells were transfected with Tax expression vectors (1 µg): Tax WT, TD1 (Δ1–37), TD55 (Δ55–92), TD99 (Δ99–142), TD150 (Δ150–198), TD254 (Δ254–287), or TD319 (Δ319–353). **A**) Forty-eight hours post transfection, total RNA was isolated by TRIzol extraction and expression of miR326 was determined by quantiMiR PCR kit using primers specific to miR326. Values shown are normalized to U6 and shown relative to control. **B**) Primary transcripts encoding miR326 were detected by RT-qPCR against a region upstream of the miRNA hairpin. Quantities were normalized to GAPDH and efficiency of Drosha processing was determined by charting the expression of mature miRNA over the expression of the primary transcript. Data is shown with the efficiency in the control cells set to 100%. **C**) A graphical depiction of the Tax mutant constructs TD1 and TD55 with deleted regions and the corresponding domains and motifs of full length Tax. The function of each domain is indicated in brackets and arrows, as well as the associated interacting cellular proteins below each region. The proposed Drosha binding region is indicated by the grey shaded box.

We have shown that Tax and Drosha interact *in vitro* and that this complex results in a downregulation/degradation of the cellular Drosha protein. This suppression of Drosha is not completely efficient however, as there is still some endogenous Drosha remaining in HTLV-1 infected cells. Here we question whether the remaining Drosha is functional in Tax expressing cells. We utilized a system where we quantify levels of cellular miR326 in Tax transfected cells normalized to a U6 control. We then calculate Drosha functional efficiency as a measure of the relative level of miRNA present versus the relative level of primary transcript, where 100% is the rate of production in the control. To this end, we transfected 293T cells with expression vectors (1 µg) for wildtype Tax or the following iterative Tax deletion mutants: TD1 (Δ1–37), TD55 (Δ55–92), TD99 (Δ99–142), TD150 (Δ150–198), TD254 (Δ254–287), or TD319 (Δ319–353). We collected the cells 48 hours post-transfection and isolated total RNA by TRIzol extraction. The expression of miR326 was determined by the quantiMiR PCR kit and is shown as a fold change in Figure 4A normalized to U6 and relative to mock transfected control. This data indicates that the Tax D1 mutant and the TaxD55 mutant increase miR326 expression 1.5 and 4 fold, respectively, above basal levels. Interestingly, the Tax mutants spanning from residue 99 to 353 have no functional implications for miR326 expression. These mutants are as functional as the Tax WT, indicating that these regions of deletion are not critical for Drosha function. The significance of the Tax D1 and D55 mutants therefore implies that the regions of Tax deleted in these mutants are necessary for Drosha interaction as well as suppression of function. In order to compare the increase in expression of miR326 to the overall transcript levels in the cell, therefore ruling out non-specificity, we detected primary transcripts encoding miR326 against a region upstream of the miRNA hairpin. These values were normalized to GAPDH and are shown in Figure 4B as a ratio of the relative level of miRNA present over the relative level of primary transcript. The mock transfected cells were set to 100% efficiency. This data indicates an increase in Drosha efficiency of 100% over the mock transfected cells with the Tax D1 mutant. The Tax WT vector decreased Drosha functionality by 25% as compared to the control. It is interesting that the Tax D1 mutant increased the efficiency of processing of Drosha 100%, however, only increased the relative miR326 expression 1.5 fold. These two values are not necessarily proportional as the extrapolation of an active, functional Drosha in the presence of this mutant, does not necessarily have to result in a direct effect on miR326. In Figure 4C we represent a more detailed schematic of the binding domains and motifs of Tax. We depict important binding and interactive regions of Tax with brackets, including the numbered residues. We also depict the cellular proteins with which Tax interacts below each sequence of interest. We propose that Drosha is binding to the N-terminus of Tax, however, more specifically to the region of Tax from 1–92, depicted as the grey shaded area. We have previously confirmed that Tax interacts with the proteasome at residues 23–62, which is also the site of the NLS, Zn finger, as well as the domain involved in CREB activation [47]. This data agrees with the interactions of Tax and Drosha in Figure 3, where the N-terminus of Tax is responsible for Drosha binding. Collectively, these data indicate that the Drosha in Tax-containing and HTLV-1 infected cells is mostly functionally inactive and the functional suppression of Drosha is dependent on its interaction with a small region of the N-terminus of Tax.

### Loss of Drosha and DGCR8 Increases Viral Replication

**Figure 5:**
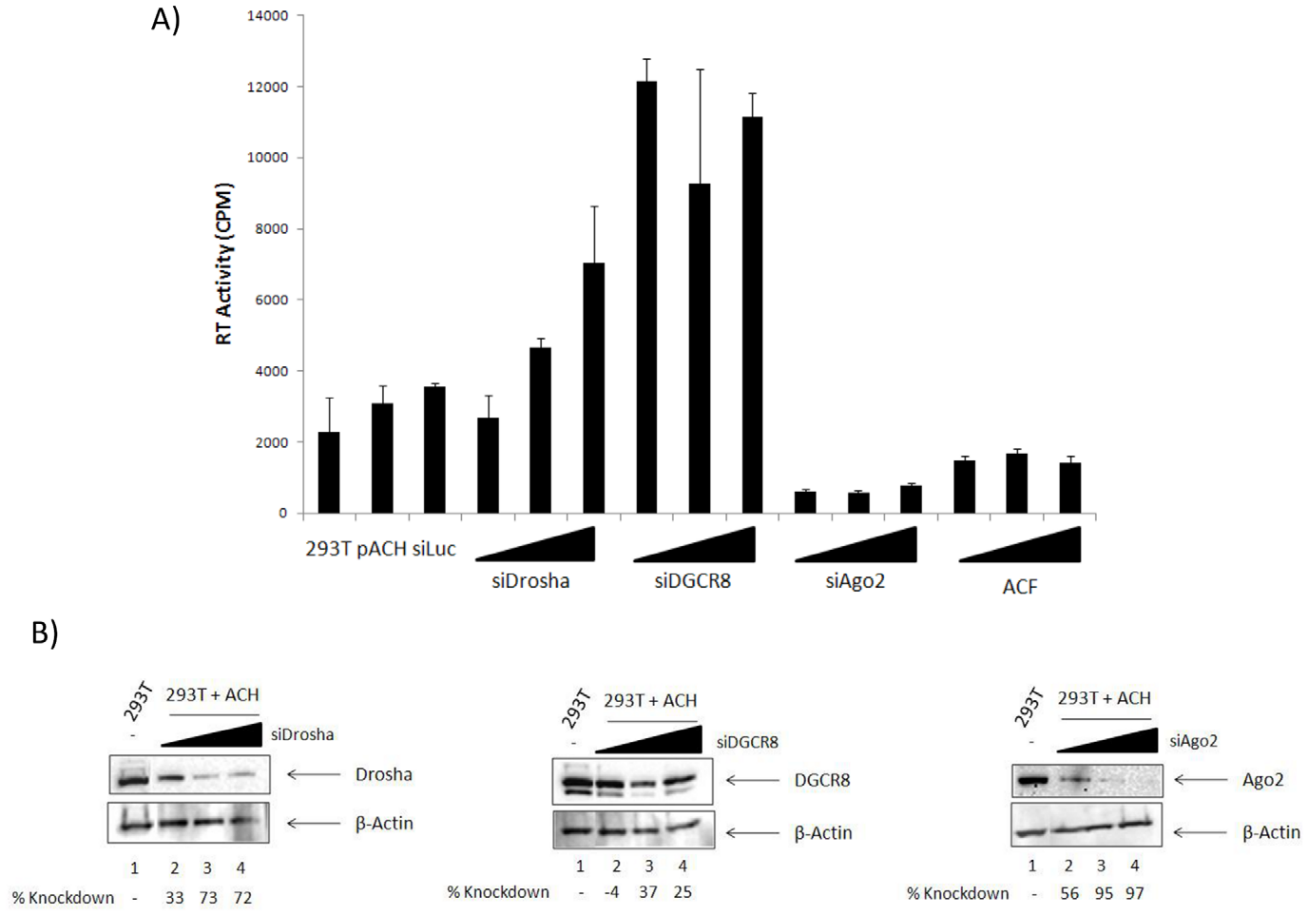
Loss of Drosha increases viral replication. **A**) 293T cells were transfected with HTLV-1 pACH (5 µg) and 24 hours later were transfected with siRNAs against Luciferase (150 nM), Drosha (50, 150, and 300 nM), DGCR8 (50, 150, 300 nM), and Ago2 (50, 150, 300 nM) or were treated with 0.1, 1.0, or 2.5 µM Acriflavine (ACF). At 72 hours post-transfection, supernatants were collected and assessed for viral replication by RT (reverse transcriptase) assay. Data was collected in triplicate from at least 2 independent experiments. **B**) 293T cells from panel A were assayed by western blot for the efficiency of siRNA knockdown of the target proteins. β-Actin serves as a positive loading control.

We have shown above that Drosha is downregulated, degraded, and mostly inactive in HTLV-1 infected cells, however, it was not clear what effect this dysregulation of Drosha would have on viral replication. Here we investigated whether loss of cellular Drosha by siRNA knockdown could affect viral replication. In doing this, it is important to distinguish between a Drosha-specific dysregulation as opposed to an overall disruption of the RNAi pathway, therefore we used siRNA against other RNAi components as a control. We transfected 293T cells with HTLV-1 infectious pACH clone (5 µg), and 24-hours post transfection, with siRNAs against Luciferase (150 nM), Drosha (50, 150, 300 nM), DGCR8 (50, 150, 300 nM), and Ago2 (50, 150, 300 nM). At 72hours post-transfection, we collected cell culture supernatants and assayed for the presence of virus using an RT (reverse transcriptase) assay. Results of such an experiment are shown in Figure 5A. Here we observed that pACH clone was able to replicate well with the highest concentration of siDrosha and even more dramatic results were obtained with siDGCR8. Interestingly, the loss of DGCR8 across all three concentrations of siRNA resulted in a consistent activation of viral replication. A knockdown of Ago2 did not result in a significant increase in viral replication. Western blot confirmation of the siRNA knockdowns are shown in Figure 5B. In order to determine whether or not the inhibition of Ago2 would result in a decrease in viral fitness and an inhibition of replication, we adopted an alternative approach and treated 293T cells transfected with pACH with the RISC/Ago2 inhibitor Acriflavine (ACF) (0.1, 1.0, and 2.5 µM) [66]. At 72 hours post transfection, we collected supernatants and assayed for the presence of virus via RT assay. We observed that that the efficient, drug inhibition of Ago2 function decreases viral replication at levels comparable to siAgo2 transfection. Collectively, these data indicate that the loss of Drosha and DGCR8 in HTLV-1 infected cells results in an increase in viral replication and its release from the cell.

### Suppression of Cellular miRNAs by HTLV-1 can be Rescued by Over Expression of Drosha and Mimicked by antagomiRs

**Figure 6:**
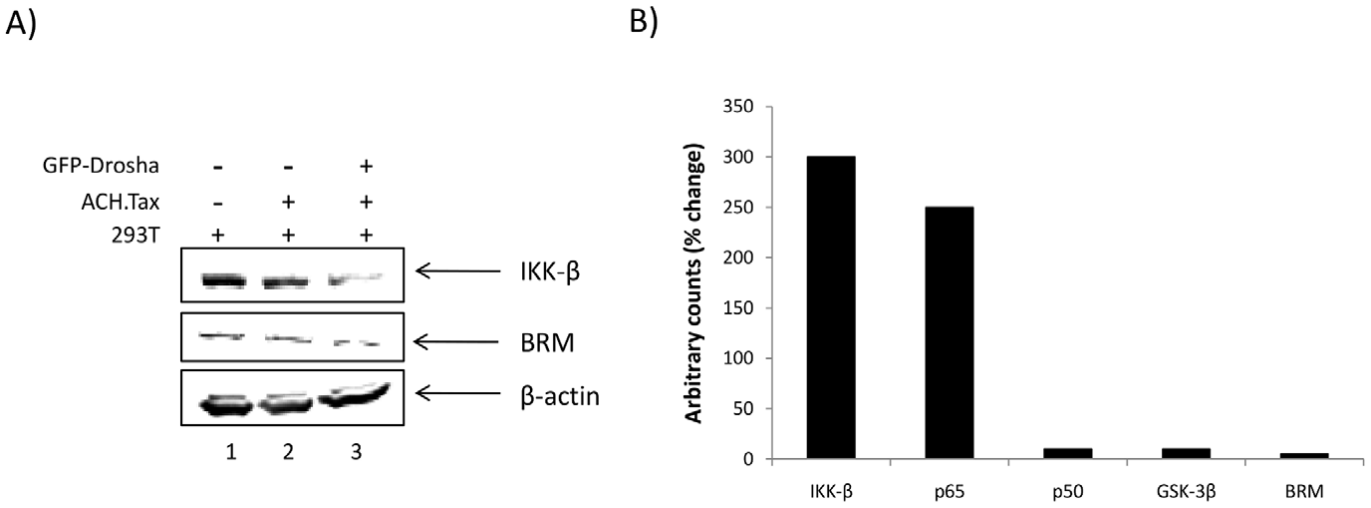
Suppression of endogenous miRNAs dysregulated by HTLV-1. **A**) 293Ts were transfected with pACH.Tax (5 µg) and 24 hours later were transfected with pGFP-Drosha (10 µg). Cells were collected 48 hours post transfection and were western blotted (50 µg) for IKK-β, BRM and β-Actin. **B**) 293T cells were transfected with Tax (5 µg) or antagomiRs (100 nM) targeting miR-199a-3p, miR-132, or Let7i. Cells were collected 72 hours post transfection, and western blotted (50 µg) for IKK-β, p65, p50, GSK-3β, BRM, and β-Actin. Densitometry counts of western blots for each indicated protein normalized to β-Actin, plotted as a percent change (measured as arbitrary counts) between antagomiR treated cells and 293T cells alone. Densitometry counts were calculated using ImageJ.

We were next interested in the regulation and downstream effects of the RNAi pathway in HTLV-1 infected cells. HTLV-1 infection results in a dramatic up (i.e. miR-130b, miR-18a, miR-20b) and downregulation (i.e. Let-7i, miR-132, miR-199a) of many host cellular miRNAs [67]. The modulation of these host miRNAs has an effect on the expression of cellular proteins such as p300, NF-κB, and BRM, to name a few, which are all recruited by Tax and play a key role in activating HTLV-1 gene transcription [5], [30]–[36], [38]–[40], [44]–[45], [56], [60], [68]–[71]. To better define the functional significance of Drosha downregulation by Tax, we transfected 293T cells with pACH.Tax (5 µg) and 24 hours later added back pGFP-Drosha (10 µg). The cells were collected 72 hours later and were lysed (50 µg) for western blots of the corresponding downstream proteins, IKK-β, BRM, and β-Actin. Here the rationale was that Tax would decrease Drosha levels, resulting in downregulation of miRNA, such as Let7i, miR-199a-3p and miR-132. Downregulation of these miRNAs would in turn effect the translation of mRNAs for genes such as IKK-β and BRM (possibly regulated by miR-199a-3p) and other proteins important for HTLV-1 gene expression. Data in Figure 6A shows that transfection of Tax downregulates both IKK-β and BRM as compared to the control Drosha add back experiment (compare Lanes 2 and 3). On the other hand, the evidence of Drosha downregulation in the presence of Tax implies the downregulation of cellular miRNAs in response to HTLV-1 infection and subsequent increase in target protein expression. Of particular interest are a set of cellular miRNAs, such as Let-7i, miR-199a-3p, and miR-132 that are downregulated after infection, resulting in an upregulation of cellular proteins that are known to be recruited and utilized by the HTLV-1 promoter. In order to mimic the effect Tax has on cells by sequestering the endogenous miR, therefore allowing for the basal, unchecked expression of these cellular proteins, we transfected 293T cells with either Tax or an antagomiR against endogenous cellular miRs which are downregulated with HTLV-1 infection. AntagomiRs used include anti-hsa-Let-7i to target p50 and p65 miRNA, anti-hsa-miR-132 to target GSK-3β miRNA, and anti-hsa-miR199a-3p to target IKK-β and BRM miRNA. Cells were collected 72 hours post transfection and western blots were performed for IKK-β, p65, p50, GSK-3β, and β-Actin. Results in Figure 6B are shown as densitometry counts of western blots for each indicated protein normalized to β-Actin, plotted as a percent change (measured as arbitrary counts) between antagomiR treated cells and 293T cells alone. These results indicate that there was a dramatic increase of protein expression of IKK-β, and p65 of approximately 300 and 250%, respectively. There was an approximate 10% increase in protein levels of p50, GSK-3β and BRM upon antagomiR treatment as compared to cells alone. Collectively, these data indicate that proteins, such as IKK-β, among others, may directly be regulated by the Tax/Drosha interaction in HTLV infected cells.

## Discussion

HTLV-1 is an oncogenic retrovirus which targets and manipulates common cellular tumor suppressors and signaling pathways in order to establish and maintain a productive infection. Recently, the role of miRNAs in both tumor development and viral infections has provided evidence that the RNAi pathway itself is also targeted by this virus. Indeed, all DNA tumor viruses, with the exception of human papillomaviruses, encode viral miRNAs which modulate tumorigenesis, therefore indicating that the cellular RNAi regulatory pathway is important for viral pathogenesis and control. For example, Epstein Barr Virus (EBV) encodes 25 viral miRNA precursors which are expressed differentially amongst various stages of latency and act to suppress chemokines, inhibit the viral DNA polymerase BALF5, and downregulate the cellular protein PUMA (p53 upregulated modulator of apoptosis) [72]–[75]. This complex interaction network between viruses and the RNAi pathway can be best described as reciprocal due to host cell miRNAs targeting both cellular and viral transcripts as well as viral miRNAs targeting both cellular and viral transcripts. Additionally, viruses can produce RNA molecules and express proteins which disrupt the cellular RNAi pathway, resulting in either induction or suppression. EBV induces the expression of the cellular miR-155 which assists in the expansion of infected B-cells and promotes their transformation whereas HIV-1 generates both RNA sequences and proteins which suppresses the RNAi pathway. Indeed, HIV-1 has been shown to not only encode its own TAR (trans-activation response region)-derived miRNA which acts as an RNAi decoy, but it also utilizes the viral transactivator Tat as an RNAi suppressor [14], [21]–[22], [76]–[80].

In this study, we characterize the effect HTLV-1 infection has on the cellular RNAi pathway. We have demonstrated that Drosha is downregulated in HTLV-1 infected cell lines, HTLV-1 transfected cells, and infected primary cells. We have also shown that Dicer is downregulated in HTLV-1 infected cell lines only, suggesting that this loss of Dicer could be due to a cell line specific phenomenon and not so much HTLV-1 infection or early stage replication.

HTLV-1 gene expression is regulated not only by Tax, but also by the viral protein Rex. Rex induces the expression of HTLV-1 structural proteins post-transcriptionally, by binding to the RxRE (Rex-responsive element) on the U3 region of the HTLV-1 LTR (long-terminal repeat) [81]–[83]. Recently, Rex has been shown to suppress the RNAi pathway by interacting with cellular Dicer, inhibiting the conversion of shRNA to siRNA [15]. As we have shown that Drosha is downregulated not only with HTLV-1 infection, but specifically in the presence of Tax, therefore we hypothesized that Tax could be interacting directly with Drosha. Tax-transfected HeLa cells show distinct, punctate foci in the nucleus when stained with antibodies against both Tax and Drosha in independent experiments. We observed that Tax and Drosha colocalize distinctly, however it is important to note that this HTLV-1-dependent downregulation of Drosha is not completely efficient, and that there is still some endogenous Drosha remaining in these cells. This is analogous and similar to the colocalization of cellular Rb and Tax in cells (Figure 2D) as we have previously shown that Tax binds directly to Rb and is a necessary interaction for the subsequent degradation of Rb *in vitro* [47]. We next demonstrated that Drosha can be found in complex with Tax at the N-terminus, specifically at the region spanning aa 1–244. Within this N-terminal region of Tax, we observed two potential motifs that could be utilized for this interaction, the Zinc finger motif and the leucine-zipper-like region. The N-terminus of Tax is most commonly known for the CREB (cAMP-responsive element-binding)-transactivation domain where the transcription factors p300/CBP, and p/CAF (p300/CBP-associated factor) bind and promote viral transcription from the LTR [56]. We have specifically shown the interaction of Drosha and Tax with an immunoprecipitation of Drosha from Tax-expressing cells in the presence of proteasome inhibitors. We indicate that this interaction is not seen when a cell is expressing the Tax mutant, ACH.M22. Interestingly, the N-terminus portion of Tax is also known to interact with the proteasome, therefore this dual binding allows Tax to bring Drosha in close proximity with the proteasome complex, resulting in the degradation of Drosha. This proteasomal degradation is confirmed by a 50% recovery of total Drosha protein levels in HTLV-1 infected cells when treated with proteasome inhibitors PSI (cbz-ile-glu-(O-t-Bu)-ala-leucinal) and ALLN (*N*-acetyl-leu-leu-norleucinal). These proteasome inhibitors have also been well documented as anticancer and antiagiogenic therapeutics. Furthermore, a number of colleagues in the field have shown that proteasome inhibitors such as PS-341 or bortezomib can in fact downregulate HTLV-1 levels, resulting in a decrease of T-cell leukemia cells [84]–[88]. This is consistent with our siDrosha and proteasome inhibitor data, where a drug/peptide inhibitor can regulate miRNA machinery by increasing proteins such as Drosha resulting in an increased host cellular RNAi response against HTLV-1 activity.

Three large studies have recently examined the dysregulation of cellular miRNAs in both HTLV-1 infected cell lines and ATLL samples, resulting in surprisingly little overlap [67], [89]–[91]. These studies do however provide a large pool of upregulated and downregulated cellular miRNAs from which to pull functional data in the context of HTLV-1 infection. We show here, that in the presence of Tax, endogenous Drosha is less functional and not as efficient in processing miRNAs. Drosha efficiency, as measured by the processing of endogenous miR326 as compared to overall levels of transcript, was highest in the presence of N-terminal Tax deletion mutants from residues 1–92. This again indicates that the interaction between Tax, Drosha, and the proteasome occurs on the N-terminal region of Tax.

We also show that in the absence of Drosha, HTLV-1 viral replication increases by at least two-fold. The knockdown of DGCR8 also resulted in a significant increase in HTLV-1 replication, suggesting a dysregulation of the microprocessing complex. The downregulation of HTLV-1 replication with Ago2 knockdown was validated with a comparable downregulation when cells were treated with the RISC inhibitor ACF.

We suggest that HTLV-1 dysregulates the RNAi pathway, including up- and down-regulation of cellular miRNAs by inhibiting the function of and degrading Drosha, resulting in a modulation of tumor and viral suppressing cellular functions. One miRNA upregulated in HTLV-1 infection, miR-146a, can be activated by Tax through an NF-κB-dependent pathway. This miRNA, however, has been experimentally shown to both interfere with HTLV-1 infected cell growth, as well as stimulate infected cell growth upon overexpression [90]. Overexpression of miR146a appears to favor the proliferation of an HTLV-1 infected cell population, however this effect is not always reproducible [67]. Due to the nature of the control of miRNA production in HTLV-1 infection, it is not surprising to observe an increase in viral replication in the absence of Drosha, due likely to the downregulation of a cellular miRNA that would otherwise suppress viral replication. In order to investigate the control of miRNA production in HTLV-1 cells, we analyzed the pool of published downregulated miRNAs for those that target cellular proteins known to interact with Tax and regulate transcription. Our rationale here was that if HTLV-1 infection downregulates a particular miRNA which targets a cellular protein necessary for Tax transactivation, we could mimic this interaction by introducing an antagomiR into uninfected cells, therefore disabling the endogenous miRNA and increasing protein expression. Based on three recent studies which examined expression changes of cellular miRNAs in HTLV-1 infected cell lines, we selected the downregulated miRNAs, miR-199a, miR-132, and miR-Let7i for further analysis [67], [89]–[90], [92]. In ovarian cancer cells, miR-199a-3p has been shown to regulate IKK-β to affect NF-κB activity as well as targeting the SWI/SNF subunit BRM in a variety of human cancers [93]–[94]. Upon KSHV (Kaposi’s sarcoma-associated herpesvirus) infection, as well as HSV-1 (herpes simplex virus-1) and HCMV (human cytomegalovirus) infection, miR-132 is upregulated and regulates the transcriptional co-activator p300 [95]. Following microbioal infection of *Cryptosporidium parvum,* miR-Let7i forms an inducible silencing complex with the NF-κB p50 subunit [96]. Confirming this rationale in the scope of HTLV-1 infection, we observed a increase in IKK-β and BRM protein levels in the presence of Tax and an opposite effect upon overexpression of functional Drosha. We observed an increase in cellular levels of IKK-β, p65, p50, GSK-3β, and BRM upon treatment with antagomiRs against the above downregulated miRNAs as compared to cells alone, indicating that the antagomiRs mimic the cellular protein expression seen in Tax-expressing cells. Tax plays a major role in the activation of cellular NF-κB, however, HTLV-1 infected cells also develop Tax-independent activation of NF-κB. This delicate balance between activation and suppression of these cellular genes via the manipulation of the RNAi pathway by HTLV-1 infection strongly supports the notion that dysregulation of Drosha and other core components of the RNAi machinery control the rate of infection and T-cell transformation.

## Materials and Methods

### Cell Culture

Uninfected T-cell lines H9, Jurkat, and CEM cells as well as HTLV-1 infected, Tax-positive T-cell lines C81, MT2, and MT4 were obtained from the NIH AIDS Research and Reference Reagent Program. HTLV-1 infected, Tax-negative cell lines were a generous gift from Dr. Cynthia Pise-Masison (NCI/NIH, Bethesda, MD). HeLa cervical carcinoma cell line and 293T endothelial kidney cells were obtained from ATCC (Manassas, VA). Primary CD4+ T-cells (#2W-200, Lonza, Allendale, NJ) infection were a generous gift from Dr. Pooja Jain (Drexel University College of Medicine, Doylestown, PA). Suspension cell lines were maintained in RPM1-1640 media containing 10% FBS, 1% L-glutamine, and 1% streptomycin/penicillin (Quality Biological) at 37°C in 5% CO_2_. Adherent cell lines were maintained in DMEM containing 10% FBS, 1% L-Glutamine, and 1% streptomycin/penicillin (Quality Biological) at 37°C in 5% CO_2_.

### Antibodies and siRNA

Antibodies used for confocal microscopy are as follows: α-BRG1 (sc-10768), α-Rb (sc-74562), and α-GIT2 (sc-5416) were obtained from Santa Cruz Biotechnology, Inc. (Santa Cruz, CA), α-Drosha (ab12286) was obtained from Abcam (Cambridge, MA), α-Tax was generated in house, and AlexaFluor 488 Goat-anti-Rabbit (A11008), AlexaFluor 660 Donkey-anti-Rabbit (A21083), and AlexaFluor 568 Goat-anti-Mouse (A11004) were obtained from Invitrogen (Carlsbad, CA). Antibodies used for western blotting are as follows: α-Drosha (ab12286), α-DGCR8 (ab82876), α-Dicer (ab14601), α-Ago2 (ab57113), and α-Ago1 (ab5070-100) from Abcam (Cambridge, MA), α-Tax was generated in house, α-Actin (sc-1615), α-Ubiquitin (sc-9133), α-p65 (sc-7151), α-p300 (sc-585), and α-IKK-β (sc-7329) were obtained from Santa Cruz Biotechnology, Inc. (Santa Cruz, CA), and α-GSK-3β (27C10) was obtained from Cell Signaling (Beverly, MA). SiRNA against Luciferase (D-002050-01-20) was obtained from Thermo Scientific (Rockford, IL) and siRNA against siDrosha (GS29102), siDGCR8 (GS54487), and siAgo2 (GS27161) were obtained from Qiagen (Valencia, CA).

### Electroporations/Transfections

293T cells were transfected with plasmid DNA according to Attractene lipid reagent recommended protocols (Qiagen, Valencia, CA). Briefly, 293T cells at 2.5×10^5^ cells were plated in DMEM ^+/+/+^ (500 µl, with serum, with L-glutamine, with penicillin/streptomycin) in a 24-well plate. A reaction mixture containing pcDNA, pACH, pcTax, or pACH.M22 (0.1, 1.0, or 10 µg) plasmid DNA was incubated with Attractene reagent (3 µl) in DMEM^−/−/−^ (100 µl, without serum, without L-glutamine, without penicillin/streptomycin). The reaction mixture was incubated for 20 min at 25°C and then added dropwise onto the cells. Cells were collected 48 hours post-transfection. Lipid transfections of pcTax, pACH, and siRNA were performed according to Attractene and Lipofectamine RNAiMax (Invitrogen, Carlsbad, CA) lipid reagent recommended protocols (Qiagen, Valencia, CA). Briefly, 2.5×10^5^ cells were plated in DMEM ^+/+/+^ (500 µl) in a 24-well plate. A reaction mixture containing ACH DNA (5 µg) was incubated with Attractene reagent (3 µl) in DMEM ^−/−/−^ (100 µl). The reaction mixture was incubated for 20 min at 25°C and then added dropwise onto the cells. At 24 hours post-transfection, a reaction mixture containing siLuc (150 nM), siDrosha (50, 150, 300 nM), siDGCR8 (50, 150, 300 nM) or siAgo2 (50, 300 nM), was incubated with RNAiMax reagent (1.5 µl). The reaction mixture was incubated for 20 min at 25°C and then added dropwise onto the cells. Lipid transfections of the Tax expression vectors (Tax WT, TD1 (Δ1–37), TD55 (Δ55–92), TD99 (Δ99–142), TD150 (Δ150–198), TD254 (Δ254–287), or TD319 (Δ319–353)) were performed in 6-well plates according to Lipofectamine 2000 recommended protocols (Invitrogen, Carlsbad, CA). Lipid transfections of pACH.Tax (5 µg), pACH.M22 (5 µg), and pGFP-Drosha (10 µg) were performed with Attractene as indicated above. The wild-type pEGFP-C1_Drosha expression vector was kindly provided by Dr. Bharat Ramratnam (Warren Alpert Medical School, Brown University, Providence, RI).

### Confocal Microscopy

HeLa cells were grown on coverslips for 48 hrs post pcTax transfection. Cells were fixed with 4% paraformaldehyde for one hour. Cells were permeabilized with Triton X-100 in PBS (0.5%) for 20 min and washed with PBS without Ca^2+^ and Mg^2+^. Cells were blocked at 25°C for 10 min with Goat Serum (3%) in PBS without Ca^2+^ and Mg^2+^. Cells were incubated with primary antibody (1∶200) in Goat Serum (3%) for one hour, in the dark, at 37°C. Cells were washed 3× with PBS without Ca^2+^ and Mg^2+^ and secondary antibodies were added (1∶50) in Goat Serum (3%) for one hour, in the dark, at 37°. Cells were washed 3× with PBS without Ca^2+^ and Mg^2+^ and a sufficient volume of DAPI working solution was added to completely cover the sample, incubating for 2–10 min, in the dark, at 25°C. Coverslips were washed to remove excess DAPI and mounted onto a slide with Fluoromount G (10 µl, Southern Biotech, Birmingham, AL). Cells were imaged with a Zeiss LSM 710 Confocal System (George Washington University, Center for Microscopy and Image Analysis).

### Western Blotting

Cells were collected from culture and spun at 1,800 rpm, for 5 min, at 4°C, and pellets were washed twice with phosphate buffered saline (PBS) without Ca2+ and Mg2+ (Quality Biological). Cell pellets were resuspended in lysis buffer (50 mM Tris-HCl, pH 7.5, 120 mM NaCl, 5 mM EDTA, 0.5% NP-40, 50 mM NaF, 0.2 mM Na3VO4, 1 mM DTT, one complete protease cocktail tablet/50 mL) and incubated on ice for 20 min, with gentle vortexing every 5 min. Cell lysates were centrifuged at 10,000 rpm for 10 min. Protein concentrations were determined using Bradford protein assay (Bio-Rad, Hercules, CA). Cell extracts were loaded (50–75 µg) on a 4–20% SDS-PAGE gel, run at 200 V, and transferred onto nitrocellulose membranes. Membranes were blocked with PBS containing 0.1% Tween-20 and milk (5%), and incubated overnight at 4°C with the appropriate primary antibody. Membranes were incubated with the appropriate secondary antibody and developed next day. Whole cell extracts from CD4^+^ primary T-cells were prepared using M-PER Mammalian Protein Extraction Reagent (Pierce, USA). Cell debris was cleared from the lysates by centrifugation (5 min, 14,000 g). Protein concentrations were determined using Bradford protein assay (Bio-Rad, Hercules, CA). Cell lysates were resolved by SDS-PAGE and transferred to a PVDF membrane. The blots were blocked for 1 h with Odyssey blocking buffer (Licor, Lincoln, NE) and rinsed with PBST (PBS +0.05% Tween-20), once for 10 min and twice for 5 min. The membranes were then incubated with either α-Drosha (1∶1000) or α-Actin (1∶2000) monoclonal antibodies (Abcam, Cambridge, MA), for 2 hours at room temperature, rinsed with PBST (once for 10 min and twice for 5 min), and incubated for 1 hour with an α-rabbit and α-mouse secondary antibody (Licor, Lincoln, USA) conjugated to IRDy800 and IRDy680 respectively. Signals were detected by Licor Pearl Pulse Imager. Densitometry was performed using ImageJ software (Wayne Rasband, NIH, USA).

### Proteasome Inhibitor Treatments

C81 HTLV-1 infected cells were plated at a density of 1×10^6^ cells/well in a 12-well plate. Cells were treated with three concentrations (0.1, 1.0, and 10 µM) of the proteasome inhibitors ALLN (208750), and PSI (539160) obtained from EMD4Biosciences (San Diego, CA). Cells were collected 48 hours post treatment, washed, lysed, and Western blotted for Drosha and β-Actin. Alternatively, 293T cells were treated with 10 µM of PSI and 24 hours later were transfected with the indicated expression vectors (pACH.Tax or pACH.M22). Cells were collected, washed, lysed, and prepared for immunoprecipitation.

### RT-PCR

Total RNA was isolated from the indicated cell types using the TRIzol protocol (Invitrogen, Carlsbad, CA). From total RNA, 2 µl of RNA was used for the cDNA synthesis reaction using iScript (Bio-Rad, Hercules, CA). The newly synthesized cDNA was used as the template for a PCR reaction for Drosha (TGCAACTGGTAGCCACAGAG, ACACTGCTGAAGCTGGGATT) and GAPDH (GGAAGGTGAAGGTCGGAGTCAA, CCTTGACGGTGCCATGGAAT). Expression of miR326 was determined by the quantiMiR PCR kit (SBI, Mountainview, CA) using the miR326 detection (sense) primer: CCTCTGGGCCCTTCCTCCAG. Primary transcripts encoding miR326 were detected by RT-qPCR (CACCAACATCCTAGCCCAAACG, AAGTGAGAACCGGGAAGCAAG).

### Immunoprecipitation

Cell extracts from 293T cells alone, cells transfected with pACH.Tax (5 µg), or pACH.M22 (5 µg) were collected as described above. Whole cell extracts (∼1 mg) were incubated overnight, rotating, at 4°C with α-IgG, α-Drosha, or α-Tax. The next day, 50 µl of a 30% slurry of Protein A+G beads (Calbiochem, Rockland, MA) was added to the IPs and incubated for two hrs, rotating, at 4°C. The IPs were spun briefly and beads were washed 1× with TNE_150_+0.1% NP-40, followed by a 1× wash with TNE_50_+0.1% NP-40. Proteins were eluted off of the beads with Laemmli buffer, run on a gel, and western blotted for both Drosha and Ubiquitin.

### Reverse Transcriptase Assay

Supernatants were collected at indicated time points to test for the presence of virus. Viral supernatants (10 µL) were incubated in a 96-well plate with a RT reaction mixture containing 1× RT buffer (50 mM Tris–HCl, 1 mM DTT, 5 mM MgCl_2_, and 20 mM KCl), 0.1% Triton-X100, poly(A) (1 U/mL), poly d(T) (1 U/mL), and [^3^H]TTP. The mixture was incubated overnight at 37°C, 5 µl of the reaction mix was spotted on a DEAE Filtermat paper, washed four times with 5% Na_2_HPO_4_, three times with water, and then dried completely. RT activity was measured in a Betaplate counter (Wallac, Gaithersburg, MD). Data is shown as cpms (counts per minute).

### GST-Pulldown

HeLa whole cell extracts (1 mg) were prepared and incubated with 10 ug of the following GST-Tax constructs bound to glutathione-sepharose beads: GST-Tax wildtype, GST-Tax 1–244 (N-terminus), GST-Tax 244–336 (C-terminus), and GST-Tax 288–353. Extracts were brought up to 500 µl in TNE_50_+0.1% NP-40 (100 mM Tris-HCl, pH 7.5; 50 mM NaCl; 1 mM EDTA; 0.1% NP-40) buffer and incubated overnight at 4°C with rotation. The following day, samples were spun and washed twice with TNE_150_+0.1% NP-40 (100 mM Tris, pH 8.0; 300 mM NaCl; 1 mM EDTA, 0.1% NP-40) buffer and 1× with TNE_50_+0.1% NP-40 to remove nonspecifically bound proteins. Samples were loaded and run on a 4–20% Tris-Glycine SDS-PAGE gel and subjected to Western blotting for the presence of Drosha.

### AntagomiR Experiments

AntagomiRs were obtained from Qiagen as follows: miScript miRNA inhibitor Anti-hsa-let-7i (Catalog # MIN0000415), Anti-hsa-miR-199a-3p (Catalog # MIN0000232), and Anti-hsa-miR-132 (Catalog #MIN0000426). AntagomiRs were transfected into 293T cells using Attractene as described above. Briefly, 293T cells were plated at 1×10^5^ cells/well in a 6 well plate. AntagomiRs were incubated in DMEM ^−/−/−^(100 µl, 100 nM) with Attractene (1.5 µl) at 25°C for 20 min. The transfection mix was added to the cells dropwise. Cells were collected at 72 hrs post transfection, lysed, and extracts (50 µg) were loaded onto a 4–20% SDS-PAGE gel. Western blots were performed using antibodies against p65, p300, IKK-β, and β-Actin. Densitometry was performed using ImageJ software (Wayne Rasband, NIH, USA).
